# Supplementary figures and images for: Pilot Study Showing Feasibility of Phosphoproteomic Profiling of Pathway-Level Molecular Alterations in Barrett’s Esophagus
Source: Genes (Basel). 2022 Jul 7;13(7):1215. doi: 10.3390/genes13071215 (PMC9325186; doi:10.3390/genes13071215)

PCA:  
Proteomics

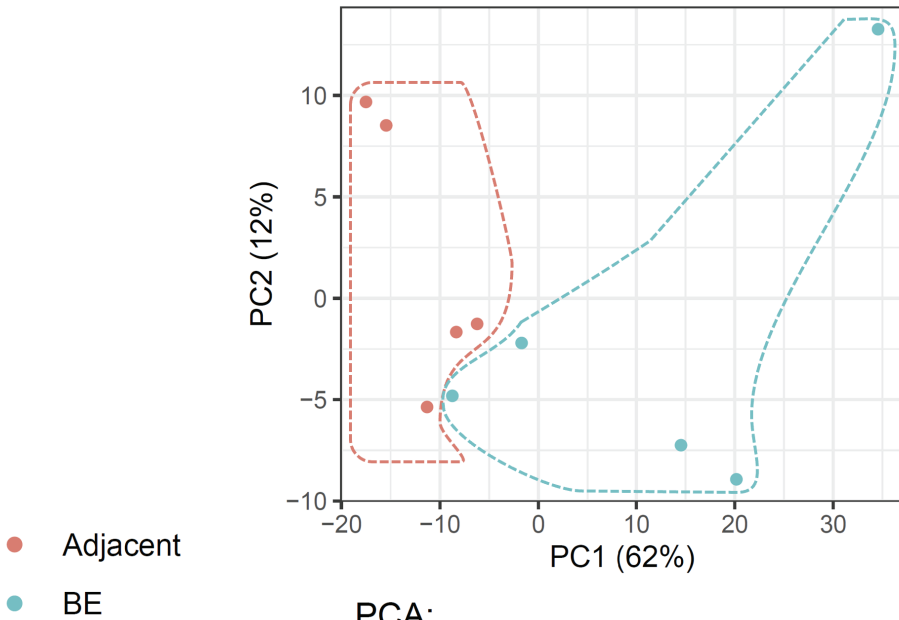

PCA:  
Phospho

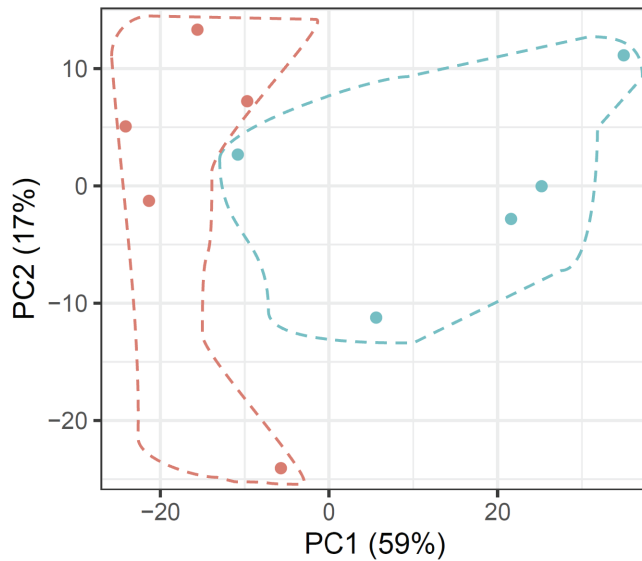

Supplement: Supplementary file 1 [file genes-13-01215-s001.zip › Supplementary Figure S1.pdf]
